# Continuous spatially informed microenvironment programs show cross-cohort concordance and context-dependent spatial organization in diffuse large B-cell lymphoma

**DOI:** 10.3389/fonc.2026.1919412

**Published:** 2026-10-01

**Authors:** Junwei Yang, Han Li, Liuxin Jiang, Lishuo Deng, Yuetong Tan, Jianxiong Zhu, Jifan Li, Yao Wang, Gaojie Liu

**Affiliations:** 1 Guangzhou Institute of Cancer Research, The Affiliated Cancer Hospital, Guangzhou Medical University, Guangzhou, Guangdong, China; 2 Medical Oncology Department, The Affiliated Cancer Hospital, Guangzhou Medical University, Guangzhou, Guangdong, China; 3 Third Ward of Gastrointestinal Surgery, The Affiliated Cancer Hospital, Guangzhou Medical University, Guangzhou, Guangdong, China

**Keywords:** continuous programs, diffuse large B-cell lymphoma, sensitivity analysis, spatial transcriptomics, tumor microenvironment, UCell

## Abstract

**Background:**

Spatial transcriptomics resolves tissue organization in diffuse large B-cell lymphoma (DLBCL), whereas most clinically annotated cohorts remain bulk transcriptomic datasets.

**Methods:**

We evaluated six literature-informed curated composite programs across bulk, single-cell and spatial datasets without assuming a discrete ecosystem taxonomy. The programs comprised Macrophage-rich, T cell-inflamed, Antigen-presentation, Stromal/fibrotic, Immune-cold/exclusion and Proliferative/cycling, with 22 memberships per program (132 memberships, 121 unique genes and 11 shared memberships).

**Results:**

In 498 GSE31312 tumors, principal components 1 and 2 (PC1 and PC2) explained 48.6% and 20.8% of score variance. No candidate discrete taxonomy demonstrated sufficient stability for retention; k=2 met silhouette and size criteria but failed both bootstrap-stability thresholds. Historical 15-edge concordance was high for GSE10846 (rho=0.936) and GSE181063 (rho=0.982), whereas the most restrictive GSE10846 edge-exclusion sensitivity, removing both shared-gene and proliferation-associated edges, reduced rho to 0.690. Single-cell analysis of 14,368 DLBCL cells from four patients and eight samples provided descriptive lineage context. In Visium V2 areas, the antigen-presentation series was treated as AP-residual (HLA-unmeasured) because only 13/22 program genes were measurable and all nine HLA genes in the defined program were unavailable. Matched-random-gene-set support was broad but not universal (24/30 DLBCL, 18/24 context/background, 42/54 overall; 25/32 primary-plus-exploratory), whereas both depth-adjusted residual endpoints were supported in 30/30 DLBCL area-program combinations.

**Conclusion:**

These findings support continuous program-level interpretation while retaining explicit provenance, stability, assay, method and sampling limitations.

## Introduction

Diffuse large B-cell lymphoma (DLBCL) is biologically heterogeneous. Cell-of-origin (COO) and genomic classifications capture important tumor-intrinsic variation (1–5), but immune, stromal and proliferative transcriptional features also vary across tumors and tissue regions (6–11). Spatial, single-cell and digital-pathology studies have provided increasingly detailed views of this microenvironmental organization (8–11), while most large outcome-annotated cohorts remain bulk transcriptomic datasets without spatial coordinates.

Transferring spatially informed biology into bulk cohorts requires careful control of the estimand. Bulk gene-set scores are abundance- and state-weighted transcriptomic measurements; they do not reconstruct spatial architecture, estimate direct cell fractions or demonstrate cellular interactions. Correlated program scores may form continuous axes rather than stable classes, and incomplete gene detection in spatial data can alter score interpretation. These issues motivated a framework centered on continuous programs, explicit model-form adjudication and sensitivity analysis.

We evaluated six literature-informed curated composite programs across GSE31312, GSE10846, GSE181063, GSE182434 and GSE276542. We first characterized COO-associated variation and low-dimensional geometry in GSE31312, then evaluated candidate k values from 2 to 6 using criteria defined during revision before execution of the revised clustering analysis. Because no candidate showed sufficient stability for retention, downstream inference used continuous scores and principal components 1 and 2 (PC1 and PC2) rather than class labels or centroids. External cohorts assessed structural concordance, single-cell data provided descriptive lineage context, and spatial data assessed primary UCell distributions, spatial statistics, matched-random-gene-set and depth-adjusted residual sensitivities, and restrained ligand-receptor expression support.

## Materials and methods

### Study design and datasets

This retrospective study used public datasets. GSE31312 (n=498; activated B-cell-like [ABC]=214, germinal-center B-cell-like [GCB]=237, unclassified [UC]=47) was the discovery cohort for continuous program scoring, COO comparison, principal-component analysis and candidate-k adjudication (12). GSE10846 comprised 414 clinical biopsies plus six sorted CD19-positive or CD19-negative profiles; the archived historical structural-concordance benchmark used all 420 expression profiles, whereas newly harmonized scoring and orthogonal sensitivity analyses used the 414 unsorted clinical biopsies (6). For GSE181063, the GEO Summary/Overall Design describes 1,311 patients/cases, whereas the public sample inventory and the downloaded expression and metadata matrices contain 1,310 profiles; all 1,310 available profiles were used here, and no additional project-level exclusion was inferred (13, 14). GSE182434 contained 28,416 cells in total, including 14,368 DLBCL, 10,219 follicular lymphoma and 3,829 tonsil cells; primary single-cell contextualization used the 14,368-cell DLBCL subset from four patients and eight samples (8). GSE276542 provided nine capture areas: Cap.area3-Cap.area7 were the five primary DLBCL areas, whereas Cap.area1, Cap.area2, Cap.area8 and Cap.area9 were context lymphoid areas (15). All datasets were publicly available and de-identified; no new participant recruitment or intervention was performed, and additional institutional review board approval was not required. Dataset roles and cohort composition are summarized in **Supplementary Table 1**.

### Composite programs, provenance and scoring

The six literature-informed curated composite programs were Macrophage-rich, T cell-inflamed, Antigen-presentation, Stromal/fibrotic, Immune-cold/exclusion and Proliferative/cycling (9–11). Each contained 22 memberships, yielding 132 memberships, 121 unique genes and 11 shared memberships in two overlap families. The direct-source core was defined as exact genes explicitly listed in the cited source supplementary table or data; phenotype-only support and author-curated marker extensions were excluded. Direct-source-core counts were 17, 15, 8, 16, 0 and 9, respectively; author-curated counts were 5, 7, 14, 6, 19 and 13, with three additional phenotype-supported memberships in Immune-cold/exclusion. Because its direct-source core contained zero genes, the Immune-cold/exclusion direct-source sensitivity was structurally non-evaluable and no substitute signature was introduced. Shared memberships comprised *CXCL9* and *CXCL10* between T cell-inflamed and Antigen-presentation, and *ACTA2*, *COL1A1*, *COL3A1*, *CXCL12*, *FN1*, *LOX*, *MMP2*, *MMP9* and *PDGFRB* between Stromal/fibrotic and Immune-cold/exclusion. Missing genes were never substituted or imputed. The primary GSE31312 analysis used an archived historical single-sample gene set enrichment analysis (ssGSEA)-derived score matrix (16, 17). Each program score was standardized across the 498 GSE31312 samples as z=(x−mean)/SD using the GSE31312 sample mean and sample standard deviation; archived raw and standardized score fields are retained in the supporting tables. The exact historical upstream gene set variation analysis (GSVA) package version was not recorded. However, rescoring with the specified GSVA 2.4.9 ssGSEA implementation reproduced the archived historical raw scores to machine precision (Pearson r=1 and Spearman rho=1 for all six programs; maximum absolute difference ≤5.55×10^−16^), and the standardized scores likewise agreed to machine precision (maximum absolute difference ≤1.15×10^−14^; **Supplementary Table 12I**). Thus, historical software-version provenance is incomplete, but the archived numerical score space is reproducible with the specified modern implementation. GSE182434 and spatial analyses used the recorded UCell implementations (18). Full membership-level provenance is reported in **Supplementary Tables 2A–E**; **Table 1** provides the compact program-level summary.

**Table 1:** Program-level provenance summary.

Program	Direct-source core	Author-curated	Source phenotype support	Shared	Direct-source sensitivity
Macrophage-rich	17/22	5/22	0/22	0	Evaluable
T cell-inflamed	15/22	7/22	0/22	2	Evaluable
Antigen-presentation	8/22	14/22	0/22	2	Evaluable
Stromal/fibrotic	16/22	6/22	0/22	9	Evaluable
Immune-cold/exclusion	0/22	19/22	3/22	9	Structurally non-evaluable
Proliferative/cycling	9/22	13/22	0/22	0	Evaluable

Counts are membership-specific. To preserve readability in the main text, **Table 1** summarizes provenance at the program level; the full 121-unique-gene mapping and membership-specific evidence are provided in **Supplementary Tables 2A–E** and in the machine-readable repository source-data files.

### COO comparison and continuous geometry

GSE31312 program distributions were compared across ABC, GCB and UC using Kruskal-Wallis tests. Benjamini-Hochberg (BH) correction was applied across the six programs (19), and the resulting false discovery rate (FDR) values were reported together with eta-squared estimates based on the Kruskal-Wallis H statistic. Principal-component analysis used the six standardized scores. Axis signs were treated as arbitrary; interpretation used the recorded loadings and program-PC correlations.

### Orthogonal abundance benchmarking

The six archived program scores were benchmarked against MCP-counter v1.2.0 (commit b6eac73e91c246fcff0bb1a5c68a816cd588fc48) (20) in GSE31312 (n=498), the 414 GSE10846 clinical biopsies and GSE181063 (n=1,310). Standard population-marker correlations were reported for all three cohorts. A sensitivity benchmark removed the union of all 121 program genes and required at least five retained markers per population-cohort; relationships falling below this threshold were classified as not evaluable rather than as persisted or failed. Multivariable abundance models reported adjusted R-squared and cross-validated R-squared. These analyses quantified concordance with broad immune and stromal abundance estimates but did not establish cellular origin, novelty, causal independence, prognostic utility or categorical taxonomy.

### Revision-defined candidate-k adjudication

Candidate k values from 2 to 6 were characterized using the proportion of ambiguous clustering (PAC), mean silhouette, silhouette relative to the best candidate, minimum cluster size, minimum-cluster median bootstrap Jaccard and overall median bootstrap Jaccard, with ConsensusClusterPlus used for consensus clustering (21). The four absolute thresholds themselves were defined during revision before execution of the revised k=2–6 analysis: mean silhouette >0.25, minimum cluster size ≥25, minimum-cluster median bootstrap Jaccard ≥0.60 and overall median bootstrap Jaccard ≥0.75. PAC rank and silhouette within 0.02 of the best candidate are reported as descriptive relative diagnostics rather than mandatory retention gates; no clustering metric was recomputed for this reporting classification. Retention required all four absolute criteria. Fourteen non-reference sensitivity variants were assessed without relaxing these thresholds after observing results. Additional model-form diagnostics comprised Hartigan dip tests for PC1 and PC2, Gaussian-mixture Bayesian information criterion (BIC) comparison, 10,000 eigenvalue permutations, and a Hopkins clusterability statistic calculated in the untruncated six-program standardized score space using 49 sampled points and seed 31312085. These diagnostics were interpreted jointly and did not define an additional clustering-selection rule. Failed partitions were not assigned biological names or used downstream.

### Bulk structural concordance and scoring sensitivity

Historical structural concordance compared the 15 nonredundant pairwise program-correlation edges in GSE10846 (all 420 expression profiles) and GSE181063 with the archived GSE31312 edge vector. The n=420 GSE10846 analysis was retained because it is the archived historical structural-concordance benchmark; it is not treated as the preferred clinical-biopsy population for newly generated analyses. Sign concordance was reported only as a secondary descriptive metric after excluding discovery edges with |r| ≤0.05. Newly harmonized analyses were restricted to the 414 unsorted clinical biopsies. Although the exact historical GSE31312 GSVA/package version was not recorded, rescoring with GSVA 2.4.9 reproduced the archived historical raw and standardized score values to machine precision (**Supplementary Table 12I**). The archived matrix therefore remained the primary score matrix for the main analysis, while a separately specified harmonized sensitivity used R 4.5.1 and GSVA 2.4.9 ssGSEA (alpha=0.25, normalize=TRUE, minSize=5, maxSize=500) with median probe collapse across GSE31312 (n=498), GSE10846 unsorted bulk (n=414) and GSE181063 (n=1,310) (17). Full, de-overlap and common-detected-gene scenarios were evaluated; these harmonized scores did not replace the archived primary score matrix. No external class, centroid assignment, taxonomy or clinical outcome inference was used.

### Single-cell contextualization

The recorded GSE182434 DLBCL subset was visualized using existing UMAP coordinates and retained CellType annotations in Seurat 5.5.1 (22). Program-wise standardized UCell scores were generated with UCell 2.14.0 and displayed without reannotation, marker discovery or malignant-cell inference (18). The subset contained B-cell-sorted and non-B-cell-sorted samples; cell-type proportions and threshold fractions were therefore descriptive and were not interpreted as unbiased tissue composition. Cells were recognized as nested within patients and samples, and no patient-level inferential test was performed.

### Spatial analysis and sensitivity controls

Five primary DLBCL capture areas were evaluated for six composite programs, yielding 30 DLBCL area-program combinations. Cap.area1-Cap.area3 used Visium V1 and Cap.area4-Cap.area9 used Visium V2. Spatial analyses used the deposited Space Ranger filtered feature-barcode matrices, with retained barcodes reconciled to the corresponding tissue_positions_list.csv metadata. Every barcode entering program scoring and Moran/Geary analyses mapped to in_tissue=1; no in_tissue=0 barcode was retained. No additional project-level thresholds were applied for minimum unique molecular identifier (UMI) count, minimum detected-feature count, mitochondrial fraction, molecular outliers or manual spot exclusion, and no new manual tissue mask was imposed. Thus, the analytical spot universe corresponds to the deposited Space Ranger filtered tissue-associated barcodes rather than to a newly re-filtered subset. Cap.area6 and Cap.area7 each contained 4,992 analyzed spots because all 4,992 physical positions were marked in_tissue=1 in the deposited metadata; they originated from different patients (p01 and p08, respectively). Coverage of at least 16/22 genes was primary-eligible, 12-15/22 exploratory and fewer than 12/22 not evaluable. In Cap.area3, Antigen-presentation retained 21/22 genes and lacked *HLA-B*; *LGALS9* was unavailable for Immune-cold/exclusion and *HMGB2* for Proliferative/cycling. In all Visium V2 areas (Cap.area4-Cap.area9), the antigen-presentation series retained 13/22 genes and lacked all nine HLA genes included in the defined program (*HLA-DRA*, *HLA-DRB1*, *HLA-DPA1*, *HLA-DPB1*, *HLA-DQA1*, *HLA-DQB1*, *HLA-A*, *HLA-B* and *HLA-C*); these values are therefore reported as AP-residual (HLA-unmeasured) and should not be interpreted as the complete antigen-presentation program. *CD8B* was also unavailable for T cell-inflamed in all V2 areas. No missing gene was substituted or imputed. Cap.area1, Cap.area2, Cap.area8 and Cap.area9 were retained as descriptive lymphoid-tissue comparators rather than independent disease controls because they were not prospectively matched control specimens and capture areas are analytical tissue units rather than independent patient-level replicates. Analyses used R 4.5.1, Seurat 5.5.1 and UCell 2.14.0; scoring sensitivity used GSVA 2.4.9 (17, 18, 22). Method concordance compared the primary UCell implementation with SCTransform v2 plus UCell and LogNormalize plus GSVA ssGSEA (17, 18, 23). Physical-distance calibration used the deposited Space Ranger scalefactors and the nominal 55-micrometer Visium spot diameter; calibrated 500-micrometer scale bars were included in the spatial-map displays. Spatial weights and permutation tests were implemented using project-defined edge-list functions in R. Moran’s I, Geary’s C and bivariate Moran’s I used the fixed first-order Visium adjacency graph; primary score-label tests used 9,999 permutations, with FDR correction applied within the defined analysis families (24–27). Bivariate Moran analysis followed established multivariate/local spatial-association formulations (26, 27). A targeted independent cross-check with spdep 1.4.2 (28) reproduced the univariate custom statistics within numerical precision (maximum absolute difference 2.78 × 10^−17^); bivariate Moran’s I was cross-checked using the equivalent symmetric binary listw/spatial-lag representation.

Spatial sensitivity analyses used a 54-combination universe comprising nine capture areas by six programs. Matched-random-gene-set tests used 1,000 expression/detection-matched sets. Depth-adjusted residual analyses modeled score as a function of log-transformed UMI and detected-feature counts and were interpreted separately from the matched null. Direct-source-core sensitivity used only exact source-listed genes and introduced no substitute core. Method consistency comprised 108 area-program-method comparisons. Not evaluable denoted an unavailable analysis under the stated restriction and was not interpreted as nonsignificant or absent.

### Exploratory continuous-program survival analysis

Overall survival was examined only as an exploratory analysis of the six continuous programs in GSE10846. The sample flow was 420 starting profiles, 414 with clinical endpoint/treatment metadata, 233 R-CHOP-like cases with complete six-program scores and overall survival (60 events), 182 clinical-adjusted complete cases (47 events), and 181 positive-time complete cases (46 events). Four six-test families were retained: univariate Cox, clinical-adjusted Cox, positive-time clinical-adjusted/C-index, and positive-time clinical-plus-proxy-adjusted models. Benjamini-Hochberg correction was applied within each six-test family. No class-based Kaplan-Meier, class-based Cox, cutpoint optimization or prognostic classifier was used.

### Ligand-receptor expression support

Ten pre-existing curated ligand-receptor axes were summarized using recorded same-spot expression concordance, k=6 neighborhood expression support with 1,000 permutations and separate-gene maps. No program-level ligand-receptor association was generated. These analyses did not test direct signaling, physical contact, receptor activation, functional communication or causality.

## Results

### Program provenance and COO-associated variation

The program contract contained six literature-informed curated composite programs, 22 memberships per program, 132 memberships, 121 unique genes and 11 shared memberships (**Table 1**; **Figure 1**; **Supplementary Table 2**). Direct-source-core counts were Macrophage-rich 17/22, T cell-inflamed 15/22, Antigen-presentation 8/22, Stromal/fibrotic 16/22, Immune-cold/exclusion 0/22 and Proliferative/cycling 9/22. The two overlap families were T cell-inflamed × Antigen-presentation (*CXCL9*, *CXCL10*) and Stromal/fibrotic × Immune-cold/exclusion (*ACTA2*, *COL1A1*, *COL3A1*, *CXCL12*, *FN1*, *LOX*, *MMP2*, *MMP9*, *PDGFRB*); overlap was disclosed but was not assumed to explain the observed covariance completely. Program-level gene coverage is summarized in **Supplementary Figure 1**, and complete program-gene matching, eligibility, and scoring quality-control results are provided in **Supplementary Table 3**. All 498 GSE31312 samples were retained. Program distributions differed across COO groups with BH-adjusted FDR below 0.05 for all six programs and eta-squared estimates from 0.0114 to 0.0664 (**Figure 2**; **Supplementary Table 4**). These effect sizes were small to modest; the largest corresponds to approximately 6.6% on this rank-based effect-size scale. The associations therefore describe continuous score heterogeneity and do not define ecosystem classes.

**Figure 1:**
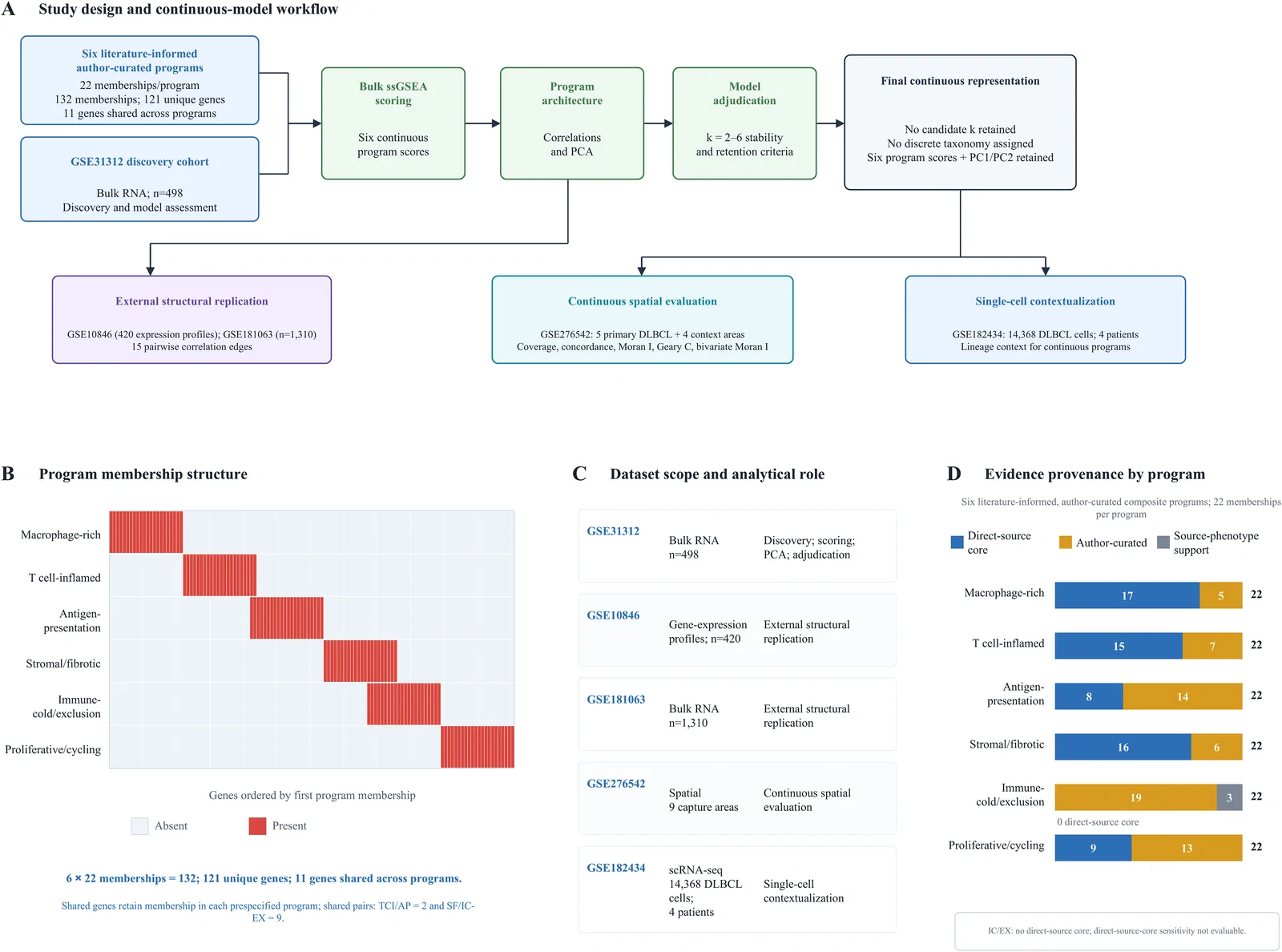
Definition and evidence provenance of the continuous program model. **(A–C)** Study design, six-program membership matrix (132 memberships; 121 unique genes; 11 shared memberships) and cohort scope. **(D)** Program-level provenance composition for the six literature-informed curated composite programs. Bars show direct-source-core, author-curated and source-phenotype-supported memberships: Macrophage-rich 17/5/0; T cell-inflamed 15/7/0; Antigen-presentation 8/14/0; Stromal/fibrotic 16/6/0; Immune-cold/exclusion 0/19/3; and Proliferative/cycling 9/13/0. Each program retains 22 memberships. Immune-cold/exclusion has no direct-source core, so its direct-source-core sensitivity is structurally non-evaluable. Bulk scores are transcriptomic single-sample scores and do not reconstruct spatial architecture.

**Figure 2:**
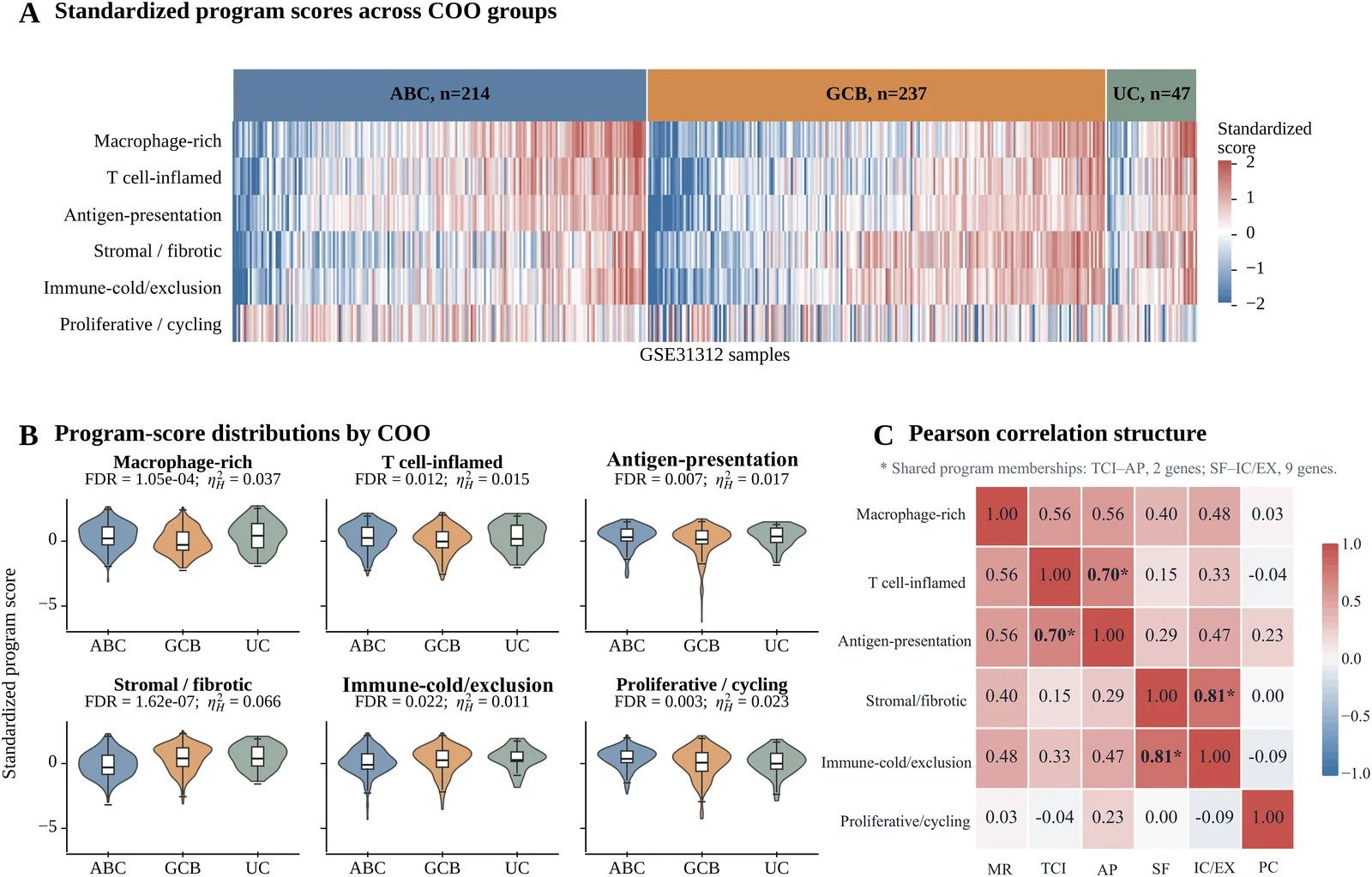
COO-associated variation and correlation structure of continuous programs. **(A)** Standardized scores across 498 GSE31312 samples grouped as ABC (n=214), GCB (n=237) and UC (n=47), ordered by ascending PC1 within COO for display. **(B)** COO distributions with Kruskal-Wallis BH-FDR and eta-squared. **(C)** Pearson correlation matrix; markers identify the two pairs with shared memberships: T cell-inflamed × Antigen-presentation (2 genes) and Stromal/fibrotic × Immune-cold/exclusion (9 genes). Shared membership does not imply that covariance is caused solely by overlap. No class, centroid or taxonomy is displayed.

In the standard MCP-counter benchmark, Stromal/fibrotic correlated most strongly with fibroblast scores in GSE31312 (r=0.829), GSE10846 (r=0.898) and GSE181063 (r=0.926). After removing all 121 program genes, the fibroblast marker set decreased from eight to three markers, below the required minimum of five, so this disjoint relationship was not evaluable. Multivariable abundance models had adjusted R-squared ranges of 0.338-0.683 in GSE31312, 0.393-0.771 in GSE10846 and 0.416-0.758 in GSE181063. In GSE10846, Proliferative/cycling was negatively associated with T-cell scores (Pearson r=-0.468; Spearman rho=-0.526), the largest absolute Pearson association for that program. Standard population-marker scores therefore explained substantial but incomplete program-score variation; the benchmark did not establish cellular origin, novelty or causal independence (**Supplementary Table 15**).

### Continuous geometry and absence of a retained taxonomy

PC1 explained 48.6% and PC2 explained 20.8% of GSE31312 score variance (**Figure 3**; **Supplementary Table 4**). PAC rank and within-best silhouette were reported as descriptive relative diagnostics and did not contribute to retention. Under the four absolute retention criteria defined during revision, k=2 met the mean-silhouette criterion (0.262 >0.25) and the minimum-size criterion but failed both bootstrap-stability thresholds: minimum-cluster median Jaccard was 0.545 versus 0.60 and overall median Jaccard was 0.669 versus 0.75. Candidates k=3–6 failed all four absolute retention criteria. Dip tests did not reject unimodality for PC1 (P = 0.804) or PC2 (P = 0.904), whereas Gaussian-mixture BIC favored two components over one (delta BIC = 30.3). Both PC1 and PC2 exceeded 10,000 permuted eigenvalue nulls (empirical P<0.001). The Hopkins statistic was 0.999535 under the prespecified implementation, indicating a strong clusterability signal under that diagnostic; however, it was interpreted jointly with the dip tests, bootstrap stability and prespecified retention criteria and was not considered sufficient by itself to retain a discrete taxonomy. These diagnostics support low-dimensional structure and a possible k=2 component structure, but not a sufficiently stable biological taxonomy. Final k was not selected and taxonomy was not assigned (**Supplementary Figure 2**, **Supplementary Table 5**).

**Figure 3:**
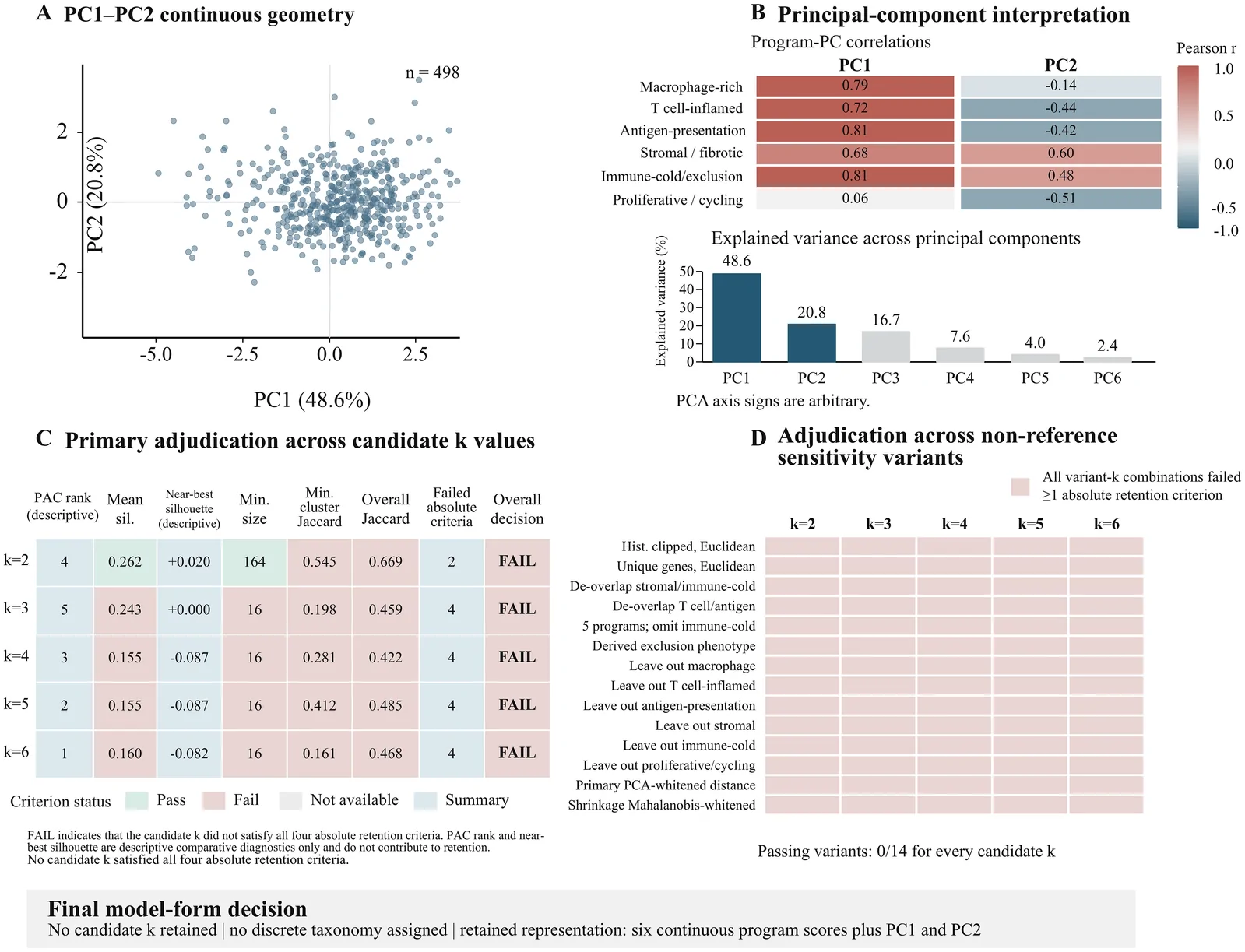
Low-dimensional geometry and revision-defined adjudication of candidate discrete models in GSE31312. **(A)** PC1-PC2 representation of 498 samples without class assignments. **(B)** Program-PC correlations and explained variance. **(C)** Candidate k=2–6 metrics. The four absolute thresholds (mean silhouette >0.25, minimum cluster size ≥25, minimum-cluster median Jaccard ≥0.60 and overall median Jaccard ≥0.75) were defined during revision before execution of the revised analysis. PAC rank and silhouette within 0.02 of the best candidate are presented as descriptive relative diagnostics rather than mandatory retention gates; no clustering metric was recomputed for this reclassification. **(D)** Fourteen non-reference sensitivity variants evaluated using the same four absolute retention thresholds. k=2 met the silhouette and size criteria but failed both bootstrap-stability thresholds; candidates k=3–6 failed all four absolute criteria. No candidate showed sufficient stability for retention. Final k was not selected and taxonomy was not assigned. The figure does not establish that discrete structure is absent or that continuity is proved.

### Bulk concordance and harmonized scoring sensitivity

In the historical primary analysis, the 15-edge correlation vector showed Spearman rho=0.936 for GSE10846 (n=420 expression profiles) and rho=0.982 for GSE181063 (n=1,310) relative to GSE31312 (**Figure 4**; **Supplementary Table 6**). Excluding only the two shared-gene edges yielded rho=0.907 in GSE10846; excluding the five Proliferative/cycling edges alone reduced concordance to rho=0.830; and excluding both the two shared-gene edges and the five proliferation-associated edges reduced it further to rho=0.690. Discovery edges with |r| ≤0.05 were excluded from sign-based interpretation, and sign agreement was treated as secondary to rank-based structural concordance. The historical GSE10846 absolute edge difference had mean |delta r|=0.154 and maximum |delta r|=0.438. In the harmonized n=414 unsorted-clinical-biopsy sensitivity, full scoring yielded rho=0.957 and 0.975 for GSE10846 and GSE181063, de-overlap yielded 0.946 and 0.950, and common-detected-gene scoring (114 unique genes; 125 memberships) yielded 0.950 and 0.979. For GSE10846, the harmonized full-program analysis had mean |delta r|=0.195 and maximum |delta r|=0.442, compared with 0.154 and 0.438 historically; harmonization therefore did not uniformly improve absolute edge agreement despite the high rank concordance. Restricting the newly harmonized GSE10846 analysis to the 414 unsorted clinical biopsies did not attenuate edge-rank concordance: rho=0.957 versus rho=0.936 in the archived historical n=420 analysis. Because the scoring implementations also differed, this comparison is interpreted as evidence that the six sorted profiles were not required to obtain high rank concordance, not as a formal leverage estimate. Thus, edge-rank structure remained substantially preserved, while absolute differences and edge exclusions qualified the strength of cross-cohort concordance. The modern sensitivity did not replace the historical primary analysis.

**Figure 4:**
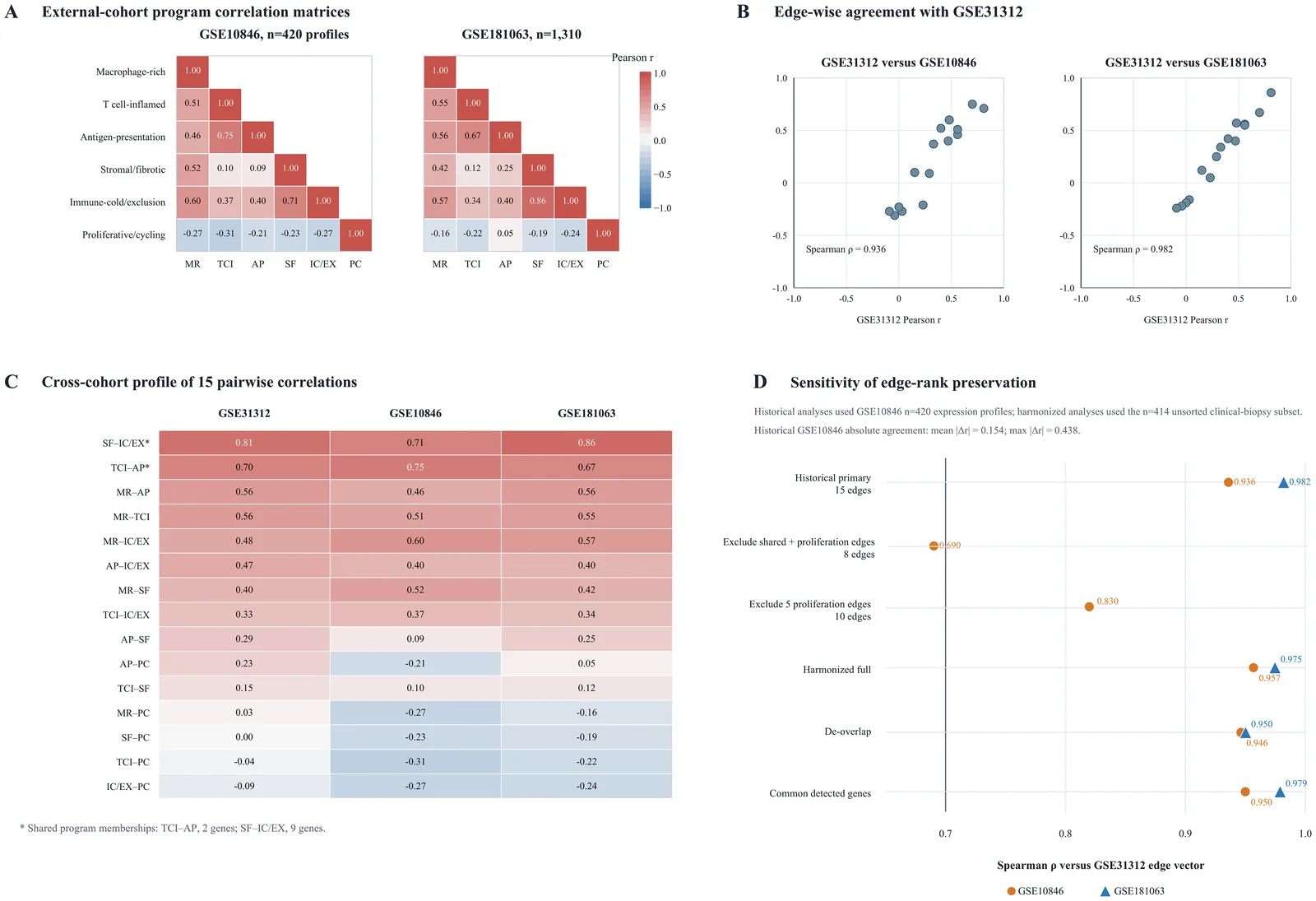
External concordance with scoring and edge-exclusion sensitivities. **(A–C)** Historical primary 15-edge analysis: GSE10846 n=420 expression profiles, Spearman rho=0.936; GSE181063 n=1,310, rho=0.982. **(D)** Sensitivity analyses. The historical n=420 GSE10846 value is retained as the archived structural-concordance benchmark; newly harmonized scoring analyses use the n=414 unsorted clinical-biopsy subset. Excluding only the two shared-gene edges yielded rho=0.907; excluding the five Proliferative/cycling edges alone yielded rho=0.830; excluding both the two shared-gene edges and the five proliferation-associated edges yielded rho=0.690. Harmonized full scoring yielded rho=0.957 and 0.975; de-overlap yielded 0.946 and 0.950; common-detected-gene scoring yielded 0.950 and 0.979 for GSE10846 and GSE181063, respectively. Historical GSE10846 absolute edge differences had mean |delta r|=0.154 and maximum |delta r|=0.438; the harmonized full-program GSE10846 values were 0.195 and 0.442, respectively, so harmonization did not uniformly improve absolute agreement. Discovery edges with |r| ≤0.05 are excluded from sign-based interpretation, and sign concordance is not used as a primary cross-cohort metric. Rank preservation was substantial, while absolute differences and edge exclusions qualify cross-cohort concordance.

### Descriptive single-cell context

The full GSE182434 dataset comprised 28,416 cells, including 14,368 DLBCL, 10,219 follicular lymphoma and 3,829 tonsil cells. The primary DLBCL contextualization used 14,368 cells from four patients, eight samples and nine retained CellType annotations (**Figure 5**; **Supplementary Figure 3**; **Supplementary Table 16**). Among these annotations, Stromal/fibrotic scores were highest in the monocyte/macrophage compartment. Fibroblast and endothelial annotations were absent, preventing direct testing of stromal-lineage localization. Because both B-cell-sorted and non-B-cell-sorted samples were included, displayed threshold fractions and cell-type proportions were not interpreted as unbiased tissue composition. The analysis was descriptive and did not infer malignant B-cell identity or validate ecosystem states.

**Figure 5:**
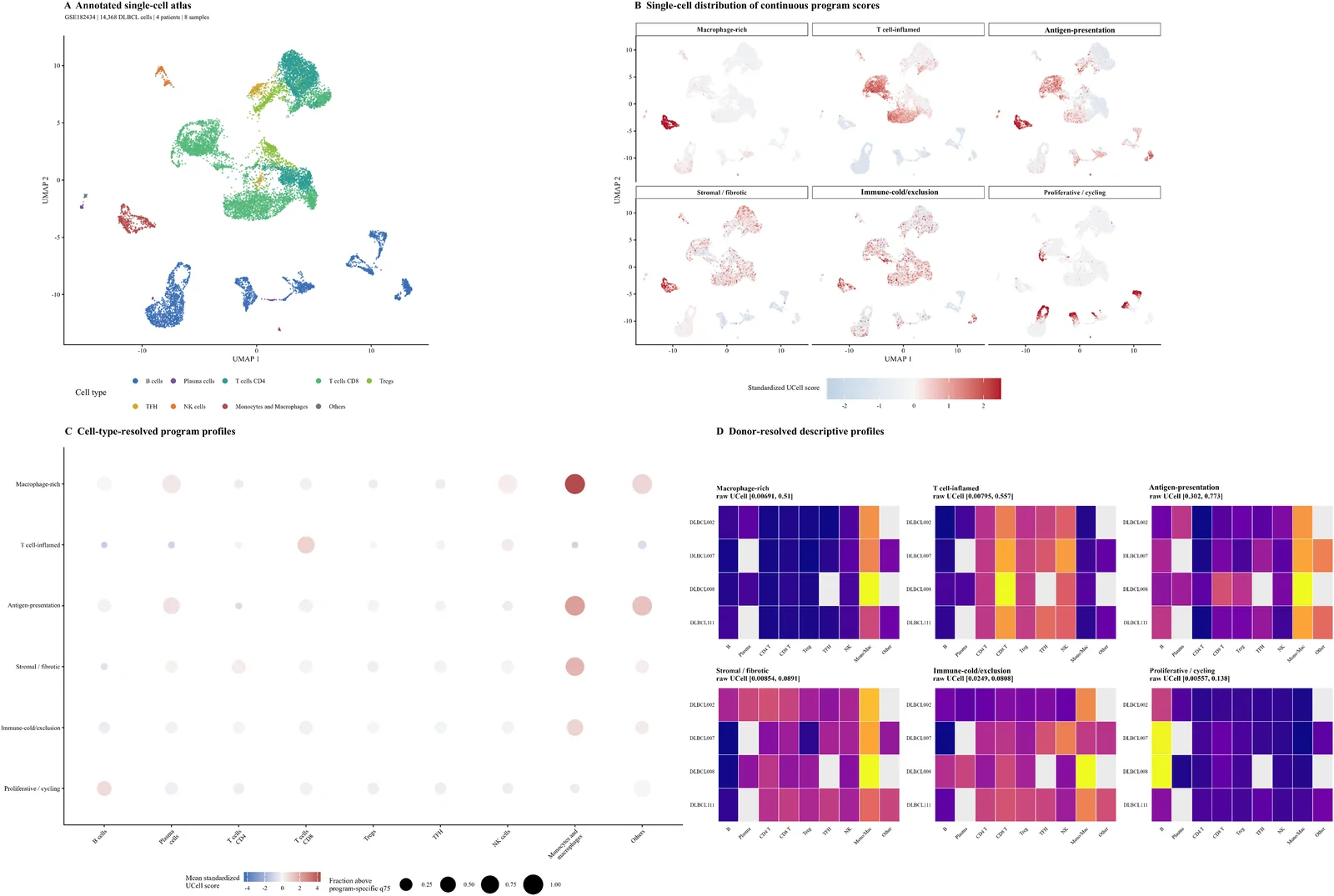
Single-cell contextualization of the six composite programs. **(A–D)** UMAP, standardized UCell overlays, annotation-resolved profiles and donor-resolved descriptive summaries for 14,368 DLBCL cells from four patients, eight samples and nine retained annotations. Among retained annotations, Stromal/fibrotic scores were highest in monocytes/macrophages. Fibroblast and endothelial annotations were absent, preventing direct testing of stromal-lineage localization. Panel **(C)** dot size is the within-annotation fraction above the program-specific 75th percentile, not tissue abundance. B-cell-sorted and non-B-cell-sorted samples are both represented. No reannotation, malignant-cell inference or taxonomy validation was performed.

### Spatial organization and sensitivity analyses

Representative UCell maps are shown in **Figure 6A**, with the complete five-area by six-program atlas provided in **Supplementary Figure 4**. Cap.area3 used Visium V1 and Cap.area4-Cap.area7 used V2. Antigen-presentation coverage was 21/22 in Cap.area3, whereas the V2 series retained 13/22 genes and lacked all nine HLA genes included in the defined program; the V2 values are therefore reported as AP-residual (HLA-unmeasured) and are not interpreted as the complete antigen-presentation program. **Figure 6B** summarizes Moran’s I across all nine capture areas: across 30 DLBCL area-program combinations, Moran’s I ranged from 0.090 to 0.928 (median 0.639), whereas across 24 context-area combinations it ranged from 0.241 to 0.802 (median 0.486). Cap.area2 tonsil showed Moran’s I of approximately 0.802 for Proliferative/cycling and 0.786 for T cell-inflamed; the approximately 0.776 value belongs to Cap.area9 tonsil V2. Spatial autocorrelation was therefore not exclusive to DLBCL tissue. Context areas were descriptive and were not treated as independent disease controls. Across the 108 method comparisons shown in **Figure 6D**, median Spearman rho was 0.878; 18/108 were below 0.7, 4/108 were below 0.5 and the minimum was 0.190 for Cap.area6 T cell-inflamed primary versus ssGSEA. Seventeen of the 18 low values involved ssGSEA; the remaining value was Cap.area3 Antigen-presentation primary versus SCTransform/UCell (rho=0.673). The Cap.area6 T cell-inflamed combination also had the lowest observed Moran’s I (0.090). We therefore regard this specific combination as weak-effect and method-unstable, and do not interpret its permutation significance as strong evidence of a reproducible biological spatial pattern. Spatial area roles, coverage, method concordance, Moran/Geary and bivariate Moran results are reported in **Supplementary Tables 7–10**.

**Figure 6:**
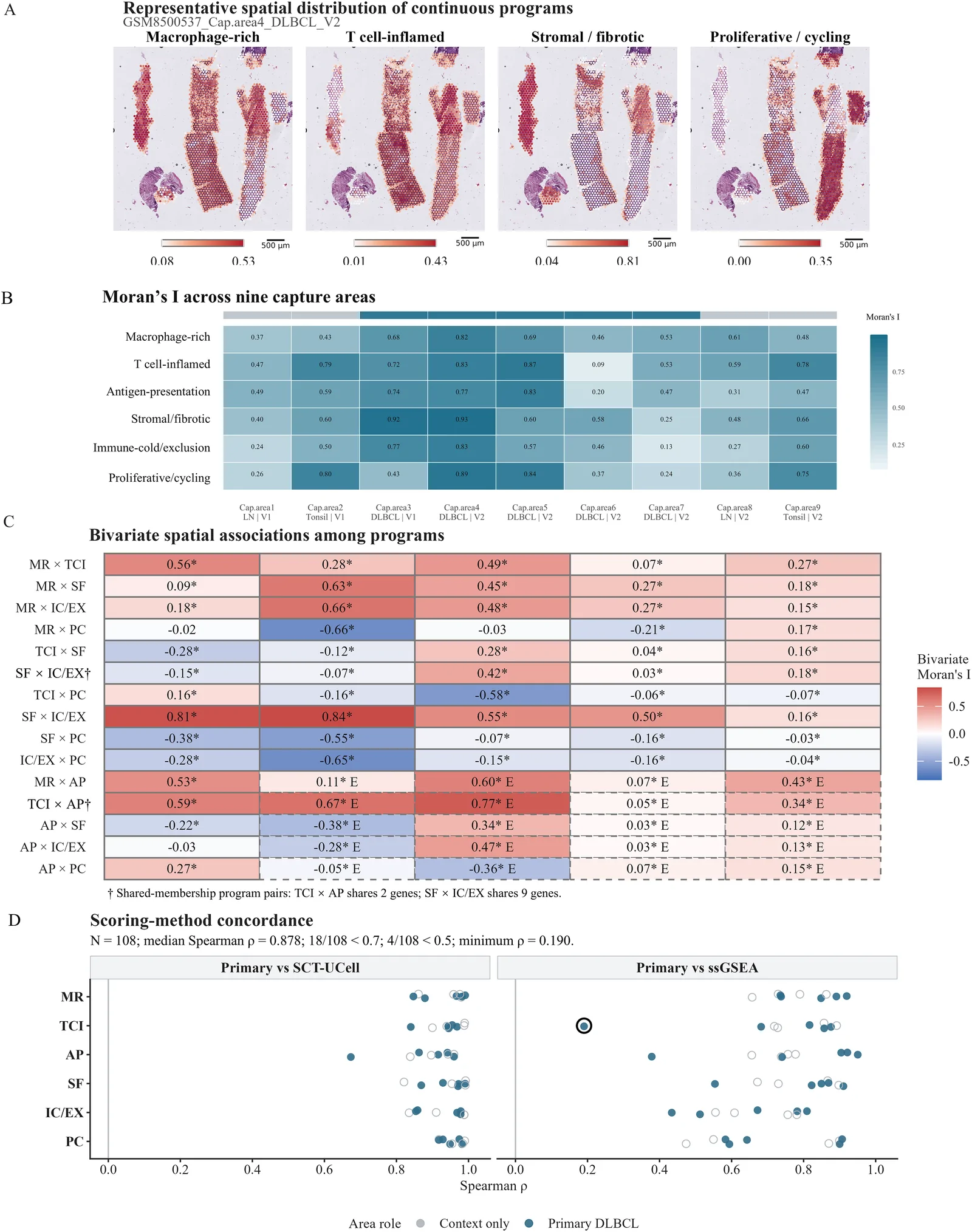
Spatial organization with context-area integration. **(A)** Representative spot-level distributions in Cap.area4 DLBCL V2 using program-specific display limits. Scale bars, 500 micrometers; each of the four spatial maps carries a calibrated bar, and all four maps are derived from the same capture area and share the same spatial coordinate system and scale. **(B)** Moran’s I across all nine GSE276542 capture areas, comprising five primary DLBCL areas (Cap.area3-Cap.area7) and four descriptive context lymphoid areas (Cap.area1, Cap.area2, Cap.area8 and Cap.area9). Context values overlap the DLBCL range and do not test tissue-class equivalence. In all Visium V2 areas, the antigen-presentation series represents AP-residual (HLA-unmeasured): 13/22 genes in the defined program were measurable, and all nine HLA genes in the program were unavailable. These values should not be interpreted as measurements of the complete antigen-presentation program. **(C)** Bivariate Moran’s I associations among program pairs across the five primary DLBCL areas. Dagger symbols (†) mark the two shared-membership pairs: T cell-inflamed × Antigen-presentation (2 shared genes) and Stromal/fibrotic × Immune-cold/exclusion (9 shared genes). Shared membership does not imply that covariance is caused solely by overlap. **(D)** Scoring-method concordance across 108 area-program-method comparisons (median Spearman rho=0.878; 18/108 below 0.7; 4/108 below 0.5; minimum 0.190; 17/18 low values involved ssGSEA). The weakest comparison, Cap.area6 T cell-inflamed primary versus ssGSEA (rho=0.190), also had Moran’s I = 0.090 and is treated as a weak-effect, method-unstable, low-confidence result rather than strong biological spatial evidence. Capture areas are analytical units and are not independent patients. Asterisks indicate BH-FDR < 0.05 within the corresponding prespecified area-specific bivariate Moran’s I testing family. ‘E’ marks the exploratory antigen-containing comparisons involving AP-residual (HLA-unmeasured) in the four primary Visium V2 areas (Cap.area4–Cap.area7).

The spatial sensitivity analyses addressed distinct questions (**Supplementary Figure 5**; **Supplementary Tables 12–14**). Matched-random-gene-set support at both Moran and Geary endpoints occurred for 24/30 DLBCL combinations, 18/24 context/background combinations, 42/54 combinations overall and 25/32 primary-plus-exploratory combinations. The seven unsupported primary-plus-exploratory combinations were Cap.area3 × Antigen-presentation; Cap.area4 × AP-residual (HLA-unmeasured); Cap.area5 × Macrophage-rich; Cap.area5 × Stromal/fibrotic; Cap.area7 × Stromal/fibrotic; Cap.area7 × Immune-cold/exclusion; and Cap.area8 × AP-residual (HLA-unmeasured). Cap.area3 × Antigen-presentation retained 21/22 genes but had Moran standardized effect -0.201 and empirical P = 0.593, showing that near-complete coverage did not guarantee matched-null support. Conversely, several HLA-unmeasured V2 AP-residual combinations exceeded the matched null, indicating that these residual scores capture spatial organization of the measurable non-HLA component but cannot be interpreted as the complete antigen-presentation program. Separately, both depth-adjusted residual endpoints were supported in 30/30 DLBCL area-program combinations. Direct-source-core sensitivity was evaluable for 21/30 primary DLBCL area-program combinations; nine were not evaluable under the direct-source-core restriction, indicating unavailable restricted scoring rather than a negative result. Matched-null, depth-adjusted and direct-source-core results were interpreted as distinct sensitivity analyses and were not used interchangeably.

### Exploratory continuous-program survival

Across 24 program-model results for overall survival, four had nominal P<0.05 and none had BH-FDR ≤ 0.05 (**Supplementary Table 12I**). The analysis sets were reported separately: 233 R-CHOP-like cases with complete program scores and overall survival (60 events), 182 clinical-adjusted complete cases (47 events), and 181 positive-time complete cases (46 events). No survival association survived multiplicity correction. These findings are exploratory/null and do not support prognostic validation, a risk classifier or clinical utility.

### Expression-only ligand-receptor support

Ten curated ligand-receptor axes were summarized in **Supplementary Figure 6**. Recorded same-spot and neighborhood metrics retained positive, negative, nonsignificant and N/E results. These observations indicate spatial expression compatibility only. They do not establish direct ligand-receptor signaling, cell-cell communication, physical contact, receptor activation or causality.

## Discussion

The central result is a change in model form. Six correlated programs and two continuous axes captured the dominant GSE31312 variation. PAC rank and within-best silhouette are comparative diagnostics rather than retention gates. Under the four absolute retention criteria, no candidate discrete taxonomy showed sufficient stability for retention. Although Gaussian-mixture BIC favored two components, at n=498 BIC can favor additional components for mildly skewed unimodal data. Likewise, PC1/PC2 eigenvalue-null exceedance was not treated as independent evidence of discreteness because the six scores share 11 gene memberships by construction. Together with the non-significant dip tests and failed bootstrap-stability thresholds, these considerations support only possible but unstable two-component structure rather than proving bimodality, continuity or a biological taxonomy.

External evidence supports qualified cross-cohort concordance of program relationships rather than class labels. Historical edge-rank concordance was high, and harmonized full, de-overlap and common-detected-gene sensitivities substantially preserved rank structure. However, absolute edge differences and the edge-exclusion analysis moderated the strength of this conclusion. Historical software-version provenance remains incomplete because the exact upstream GSVA/package version was not recorded. Importantly, this did not produce a numerical reproducibility gap: rescoring with GSVA 2.4.9 reproduced the archived historical raw and standardized GSE31312 score values to machine precision (**Supplementary Table 12I**). Downstream standardization, PCA, correlation analyses and candidate-k diagnostics are therefore reproducible from the archived score matrices, while the specified GSVA 2.4.9 implementation provides an independent numerical reproduction of the score space. The harmonized multi-cohort analysis remains a separately specified sensitivity rather than a replacement for the archived primary analysis.

The orthogonal benchmark showed that broad population scores explained substantial but incomplete program variation across all three cohorts. The strong Stromal/fibrotic-fibroblast association could not be evaluated after global gene disjointing because only three fibroblast markers remained. This benchmark does not establish cellular origin or causal independence. Similarly, the exploratory overall-survival analysis yielded no BH-FDR-significant association across 24 tests and does not support prognostic utility.

Single-cell results provide lineage context, not proof of cellular origin. Stromal/fibrotic scores were highest in monocytes/macrophages among retained annotations, but fibroblast and endothelial annotations were absent; sorting also prevents unbiased tissue-composition inference. For the published taxonomy comparison requested during review, the Cell-Eco retrieval audit examined the supplementary workbook accompanying Diaz-Herrero et al. (15), specifically **Supplementary Tables S1, S3**, and **S4**, together with the GSE276542 GEO raw files and capture-area metadata and the authors' code repository. These materials provided aggregate frequencies, differential-expression tables, raw spatial files, metadata and scripts but no deposited barcode-level Cell-Eco assignment registry. The Kotlov lymphoma microenvironment (LME) audit examined the supplementary workbook NIHMS1669159-supplement-2.xlsx and accompanying article materials, but no exact sample-level LME mapping for the retained bulk cohorts was identified. The requested direct quantitative comparisons were therefore not performed and remain a limitation. We did not infer labels from aggregate figures, reconstruct retired classifiers or train replacement classifiers, because those approaches would not constitute comparison with the authors’ deposited assignments. Our failure to retain a stable taxonomy under the present six-program framework does not invalidate published Cell-Eco or LME classifications derived using different frameworks.

Spatial capture areas are analytical tissue units rather than independent patients; Cap.area6 and Cap.area7 are from different patients (p01 and p08). All analyzed spatial barcodes mapped to Space Ranger in_tissue=1, and no additional project-level UMI, detected-feature, mitochondrial-fraction, molecular-outlier or manual spot-exclusion threshold was imposed. Spatial autocorrelation supports within-section organization but overlaps with context lymphoid tissue and is not disease-specific by magnitude alone. Antigen-presentation coverage was assay-associated: Cap.area3 V1 retained 21/22 genes, whereas V2 areas retained only 13/22 and lacked all nine HLA genes in the defined program. The V2 series is therefore interpreted as AP-residual (HLA-unmeasured), not as complete antigen-presentation biology. Coverage alone did not explain matched-null exceptions, and the 30/30 depth-adjusted result must not be substituted for 42/54 overall matched-null support. Method agreement was generally high but heterogeneous; Cap.area6 T cell-inflamed combined the lowest Moran’s I (0.090) with the weakest primary-versus-ssGSEA concordance (rho=0.190) and is therefore low-confidence for biological interpretation. Calibrated 500-micrometer scale bars are provided in **Figure 6A** and **Supplementary Figure 4** using the deposited Space Ranger scale information. Independent spdep validation reproduced the univariate custom statistics within numerical precision, with bivariate Moran’s I cross-checked against the same symmetric binary spatial-weight representation. Ligand-receptor findings remain expression-based and do not establish communication or causality.

Program provenance was also asymmetric. Direct-source-core sizes ranged from 0 to 17 genes; Antigen-presentation and Proliferative/cycling had smaller cores, and Immune-cold/exclusion had no direct-source core, making that sensitivity structurally non-evaluable. Eleven shared memberships were retained transparently, and the resulting correlations were not attributed solely to overlap. These boundaries favor program-specific interpretation over treating all six composites as equally source-grounded published signatures.

Six literature-informed curated composite programs provide a constraint-aware continuous representation of immune, stromal and proliferative variation relevant to DLBCL. Their relationships showed qualified cross-cohort concordance across independent bulk cohorts, descriptive single-cell data provided limited lineage context, and spatial analyses demonstrated non-random within-section organization with explicit context, assay, coverage, method and sampling boundaries. No candidate discrete taxonomy showed sufficient stability for retention; possible k=2 structure remained stability-limited. Final k was not selected and taxonomy was not assigned.

## Data Availability

The original contributions presented in the study are included in the article/**Supplementary Material**. The datasets analyzed in this study are publicly available through the Gene Expression Omnibus (GEO) under accession numbers GSE31312, GSE10846, GSE181063, GSE182434, and GSE276542. Versioned analysis and figure-generation scripts, metadata, and environment records are publicly available in the GitHub repository at https://github.com/Yangjunwei-GZ/DLBCL-spatial-niche-reproducibility. Machine-readable source-data files and archived reproducibility materials are publicly available in the versioned Zenodo repository, version 1.0.2 (29), at https://doi.org/10.5281/zenodo.22008715. Detailed reproducibility and data/code availability records are provided in **Supplementary Table 11**. Further inquiries can be directed to the corresponding author.

## Supplementary material

Supplementary Figure 1 Program-gene coverage across datasets and primary DLBCL spatial areas. This coverage and quality-control figure summarizes six continuous programs: Macrophage-rich, T cell-inflamed, Antigen-presentation, Stromal/fibrotic, Immune-cold/exclusion and Proliferative/cycling. Each program contains 22 memberships, for 132 memberships in total. These represent 121 unique genes because 11 genes occur in two programs. **(A)** Program-level gene coverage in GSE31312 discovery bulk transcriptomes, GSE10846 and GSE181063 external bulk cohorts, and GSE182434 single-cell contextualization. **(B)** Dataset-level totals were 132/132 (100.0%) for GSE31312, 128/132 (97.0%) for GSE10846, 126/132 (95.5%) for GSE181063 and 132/132 (100.0%) for GSE182434. **(C)** Program-gene coverage across Cap.area3-Cap.area7, the five primary DLBCL capture areas. Eligibility was defined as at least 16/22 genes for primary analysis, 12-15/22 for exploratory analysis and fewer than 12/22 for non-evaluable combinations. Antigen-presentation coverage was 21/22 in Cap.area3. In Cap.area4-Cap.area7, only 13/22 genes were measurable and all nine HLA genes included in the defined program were unavailable; these V2 rows are therefore interpreted as AP-residual (HLA-unmeasured), not as the complete antigen-presentation program. No missing gene was substituted or imputed. The panel reports coverage only and does not constitute a new biological analysis.

Supplementary Figure 2 Extended diagnostics for candidate-k model adjudication. **(A)** Consensus matrices for candidate partitions k=2-6, shown in their recorded consensus order on a common 0–1 scale. Neutral identifiers C1-Ck denote plotting partitions only and are not biological classes. **(B)** Consensus cumulative distribution function (CDF) curves for k=2-6. **(C)** CDF area and delta-area diagnostics; delta area for k=2 is structurally undefined and is displayed as not available without imputation. **(D)** PAC, mean silhouette, minimum cluster size, minimum-cluster median Jaccard and overall median Jaccard. PAC rank and silhouette relative to the best candidate are descriptive relative diagnostics and are not retention gates. **(E)** Evaluation against four absolute retention criteria: mean silhouette greater than 0.25, minimum cluster size of at least 25, minimum-cluster median Jaccard of at least 0.60 and overall median Jaccard of at least 0.75. Candidate k=2 met two of four absolute criteria (silhouette and minimum size) and failed both bootstrap-stability criteria; candidates k=3–6 failed all four absolute criteria. No candidate k satisfied all four absolute retention criteria; therefore, final k was not selected and no discrete taxonomy was assigned.

Supplementary Figure 3 Single-cell cohort composition and annotation structure in GSE182434. **(A)** Cohort boundary for the full GSE182434 dataset (28,416 cells), comprising DLBCL (14,368 cells), follicular lymphoma (10,219 cells), and tonsil (3,829 cells). Primary **Figure 5** analyses were restricted to the DLBCL component (14,368 cells from four patients and eight samples). **(B)** Descriptive cell counts across the four DLBCL patients, partitioned by B-cell-sorted and non-B-cell-sorted samples. **(C)** Composition of the DLBCL subset according to nine retained CellType annotations, shown as cell counts and percentages of the 14,368-cell subset. **(D)** CellType composition across patients. Colors denote within-patient proportions, and labels report raw cell counts. Cells are nested within patients and samples; no reannotation, marker discovery, program rescoring, or patient-level inferential test was performed for this figure. Accordingly, the observed cell-type proportions are sorting-dependent and should not be interpreted as an unbiased estimate of tissue composition.

Supplementary Figure 4 Complete primary-DLBCL atlas of six literature-informed curated composite programs. The five-area by six-program UCell atlas extends the representative spatial display in main **Figure 6** without changing scores. Cap.area3 used Visium V1, whereas Cap.area4-Cap.area7 used Visium V2. Antigen-presentation coverage was 21/22 in Cap.area3. In Cap.area4-Cap.area7, only 13/22 genes in the defined antigen-presentation program were measurable and all nine HLA genes included in the program were unavailable; these V2 panels are therefore AP-residual (HLA-unmeasured) displays and should not be interpreted as measurements of the complete antigen-presentation program. The V2 designation is written out in the figure key and legend as AP-residual (HLA-unmeasured), rather than conveyed by an unexplained exploratory marker. Scale bars, 500 micrometers; one scale bar is shown per capture-area row because all six program maps within a row share the same spatial coordinate system and scale. Program-specific display limits are shared across capture areas within each program. No rescoring was performed.

Supplementary Figure 5 Sensitivity analyses of spatial continuous-program structure. **(A)** Analysis framework and denominator structure for matched-random-gene-set, depth-adjusted residual, Moran/Geary concordance and direct-source-core sensitivity analyses. The matched-null and depth-adjustment analyses use the same 54 area-program universe: nine capture areas by six continuous programs; the residual analysis therefore comprises 108 spatial endpoints (Moran’s I and Geary’s C). **(B)** Observed Moran’s I and Geary’s C standardized effects relative to 1,000 expression/detection-matched random gene sets for 30 program-area combinations across the five primary DLBCL capture areas. Filled points denote primary-eligible combinations; outlined points denote coverage-limited V2 AP-residual (HLA-unmeasured) combinations retained as exploratory. Across the complete nine-area matched-null universe, support at both endpoints occurred for 42/54 combinations overall and 25/32 primary-plus-exploratory combinations. **(C)** Raw spatial statistics versus statistics calculated from residuals of the model program score ~ log1p(nCount_Spatial) + log1p(nFeature_Spatial). All 30 DLBCL area-program combinations had valid models, and both depth-adjusted residual endpoints had BH-FDR ≤ 0.05 in 30/30 combinations. **(D)** Concordance between Moran’s I and the spatial-departure form 1 - Geary’s C for 30 DLBCL area-program combinations. **(E)** Full-program versus direct-source-core spot-score Spearman correlations. Of 30 DLBCL area-program combinations, 21 were evaluable and nine were not evaluable under the direct-source-core restriction (N/E); N/E denotes unavailable restricted scoring, not a nonsignificant result. No missing core gene was substituted or imputed. Matched-null, depth-adjusted and direct-source-core analyses answer distinct sensitivity questions and their denominators are not interchangeable. These analyses do not establish causality, patient-level replication, pathway activity or cell-cell communication. Final k was not selected and no discrete taxonomy was assigned.

Supplementary Figure 6 Spatial expression support for curated ligand-receptor axes. **(A)** Ten curated axes were assessed across five primary DLBCL capture areas, yielding 50 possible area-by-axis evaluations; 45 were evaluable under the analysis-defined availability rule. No current continuous-program ligand-receptor association was generated. **(B)** Same-spot Spearman correlations between ligand and receptor expression, with Benjamini-Hochberg FDR retained across the 45 evaluable area-by-axis comparisons; positive, negative and nonsignificant results are shown. **(C)** k-nearest-neighbor expression support using k=6 and 1,000 permutations; color denotes log2 enrichment, dot size denotes -log10 BH FDR and asterisks denote FDR below 0.05. **(D)** Separate normalized-expression maps for *MIF*-*CD74* and *CXCL9*-*CXCR3* in GSM8500537_Cap.area4_DLBCL_V2; no composite ligand-receptor score is shown. Not evaluable means that a required ligand or receptor gene was unavailable, not that signaling was absent. These expression-based analyses do not establish same-cell interaction, physical contact, signaling, receptor activation, functional communication or causality.
